# Magnetic resonance image-based brain age as a discriminator of dementia conversion in patients with amyloid-negative amnestic mild cognitive impairment

**DOI:** 10.1038/s41598-023-49465-8

**Published:** 2023-12-18

**Authors:** Hye Weon Kim, Hyung-Ji Kim, Hyunji Lee, Hyeonsik Yang, ZunHyan Rieu, Jae-Hong Lee

**Affiliations:** 1 Research Institute, Neurophet Inc., Seoul, 06234 Korea; 2 Department of Neurology, Uijeongbu Eulji Medical Center, Eulji University, Uijeongbu, Korea; 3 Department of Neurology, Asan Medical Center, University of Ulsan College of Medicine, 88 Olympic-ro 43-gil, Songpa-gu, Seoul, 05505 Korea

**Keywords:** Alzheimer's disease, Neurodegeneration

## Abstract

Patients with amyloid-negative amnestic mild cognitive impairment (MCI) have a conversion rate of approximately 10% to dementia within 2 years. We aimed to investigate whether brain age is an important factor in predicting conversion to dementia in patients with amyloid-negative amnestic MCI. We conducted a retrospective cohort study of patients with amyloid-negative amnestic MCI. All participants underwent detailed neuropsychological evaluation, brain magnetic resonance imaging (MRI), and [18F]-florbetaben positron emission tomography. Brain age was determined by the volumetric assessment of 12 distinct brain regions using an automatic segmentation software. During the follow-up period, 38% of the patients converted from amnestic MCI to dementia. Further, 73% of patients had a brain age greater than their actual chronological age. When defining ‘survival' as the non-conversion of MCI to dementia, these groups differed significantly in survival probability (p = 0.036). The low-educated female group with a brain age greater than their actual age had the lowest survival rate among all groups. Our findings suggest that the MRI-based brain age used in this study can contribute to predicting conversion to dementia in patients with amyloid-negative amnestic MCI.

## Introduction

We are living in the era of artificial intelligence (AI), which is being widely integrated into the neuroimaging field^1^. Many AI software provide quantitative information based on brain magnetic resonance imaging (MRI) segmentation, particularly in degenerative brain disorders including mild cognitive impairment (MCI) and dementia^2,3^.

One notable aspect of AI application in neuroimaging research is the concept of "brain age." Despite debates concerning its validity and utility as a biomarker of brain health^4,5^, there is compelling evidence to suggest that brain age hold the value for predicting the risk of cognitive decline and neurological progression, including Alzheimer's disease^6–8^. The estimation of brain age can vary depending on different brain imaging techniques and algorithms employed, and there is diversity on the definition and measurement methods of brain age^9,10^.

Within this regard, the concept becomes particularly pertinent to patients with amyloid-negative amnestic MCI, who are at a risk of developing dementia^11^. The main concern in patients with MCI is whether they will be able to perform activities of daily living; in other words, whether their condition will deteriorate into dementia^12,13^. Approximately 50–60% of patients with amyloid-positive amnestic MCI develop dementia in approximately 2 years, while 10–20% patients with amyloid-negative amnestic MCI develop dementia, indicating that amyloid-negative amnestic MCI cannot be considered a simple benign condition^14,15^.

Given the implications, early detection of conversion to dementia is crucial for timely intervention and management^16,17^. To address the critical concern, our retrospective cohort study aimed to elucidate the role of brain age in predicting the conversion to dementia in amyloid-negative amnestic MCI.

## Results

### Demographic characteristics

Detailed demographic characteristics of the participants are shown in Table 1. Age, duration from onset to diagnosis, sex, education level, and occurrence of apolipoprotein E (ApoE) genotype were not significantly different between the two groups. In contrast, the K-MMSE score was significantly higher (p = 0.001) and the CDR score was significantly lower in the non-converter group (p = 0.029).

**Table 1:** Demographics and baseline characteristics of the subjects divided based on dementia conversion.

	Non-converter (N = 56)	Converter (N = 35)	p-value
Age of onset (years)	69.66 ± 9.55	72.80 ± 7.41	0.277
Age at diagnosis (years)	72.30 ± 8.88	74.69 ± 6.84	0.260
Age at MRI scan (years)	72.04 ± 9.10	74.51 ± 7.18	0.205
Duration from onset to diagnosis (months)	32.61 ± 30.50	28.37 ± 21.50	0.977
Sex (female)	29 (51.79%)	24 (68.57%)	0.174
Education (months)	9.62 ± 5.43	9.23 ± 5.53	0.681
ApoE genotype (e4 carrier%)	10 (17.86%)	6 (17.15%)	0.992
K-MMSE	26.25 ± 3.90	23.17 ± 4.20	0.001*
CDR	0.50 ± 0.00	0.54 ± 0.14	0.029*

The Student t-test was performed on normally distributed data. For continuous variables that did not show normal distributions, the Kruskal–Wallis test was performed. Group differences in dichotomous variables were evaluated using the χ^2^ test.

*MRI* magnetic resonance imaging, *K-MMSE* Korean version mini-mental state examination, *CDR* clinical dementia rating.

*p-value < 0.05.

## Volumetric results and brain age

### Correlation between neuropsychological test performance and volume of ROI

**Figure 1:**
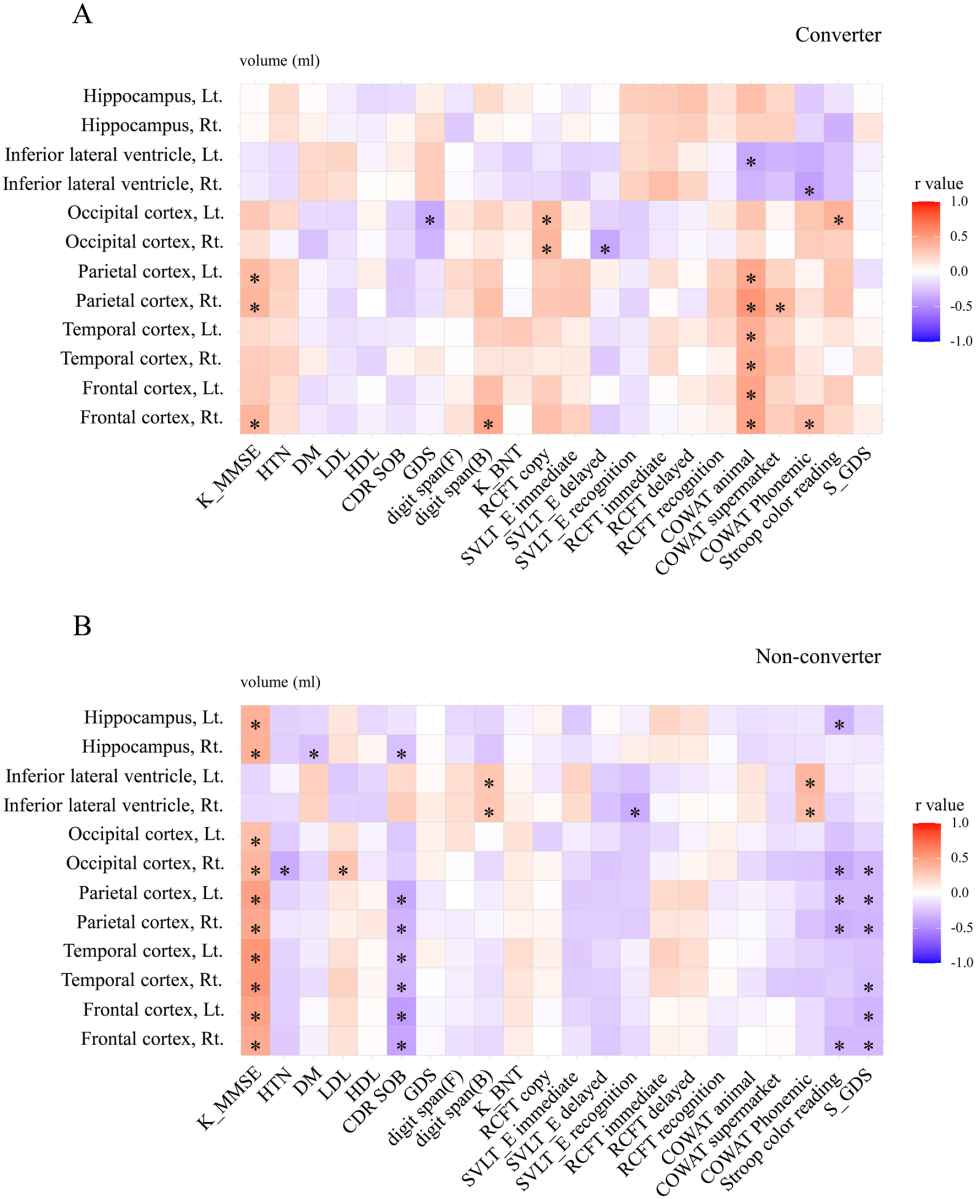
Correlation between neuropsychological test performance and volume of ROIs. A. Correlation coefficients between neuropsychologic test performance and volume of ROIs in the converter group. The converter group showed good inverse correlations between the score of the COWAT animal test and the regional volume of the left inferior lateral ventricle, and between the score of the COWAT Phonemic test and the right inferior lateral ventricle. B. Correlation coefficients between neuropsychologic test performance and volume of ROIs in the non-converter group. Interestingly, this group shows an inverse correlation between the score of the CDR-SOB and the right hippocampus, as well as between the score of the stroop color reading test and the left hippocampus. In addition, the CDR-SOB, S-GDS scores and the regional volumes of the parietal, frontal and temporal lobes were also inversely correlated. Spearman correlation test was performed. Correlation Coefficients by Spearman's rank correlation rho. Abbreviation: K-MMSE, Korean version Mini-Mental State Examination; HTN, hypertension; DM, diabetes mellitus; LDL, Low-density lipoprotein; HDL, High-density lipoprotein; CDR SOB, Clinical Dementia Rating Sum of Boxes; GDS, Global Deterioration Scale; K‑BNT, Korean version-Boston naming test; RCFT, Rey complex figure test; SVLT, Seoul verbal naming test; COWAT, controlled oral word association test; S-GDS, Short version of Geriatric Depression Scale; ROIs, regions of interest. * p-value 0.3

The volume of ROIs in patients with Alzheimer's dementia correlated well with the K-MMSE scores, especially in the non-converter group. However, the pattern of significant correlation between the results of neuropsychological test and the volume of ROIs differed between the two groups (Fig. 1). The converter group showed an inverse correlation between the Controlled Oral Word Association Test (COWAT) animal test score and the regional volume of the left inferior lateral ventricle and between the COWAT Phonemic test score and the regional volume of the right inferior lateral ventricle (Fig. 1A). Interestingly, the non-converter group showed an inverse correlation between the CDR sum of boxes (CDR-SOB) score and the regional volume of the right hippocampus and between the Stroop color reading test score and the regional volume of the left hippocampus. In addition, the CDR-SOB and GDS scores were inversely correlated with regional volumes of the parietal, frontal, and temporal lobes (Fig. 1B).

#### Comparison of brain age with the actual chronological age

**Figure 2:**
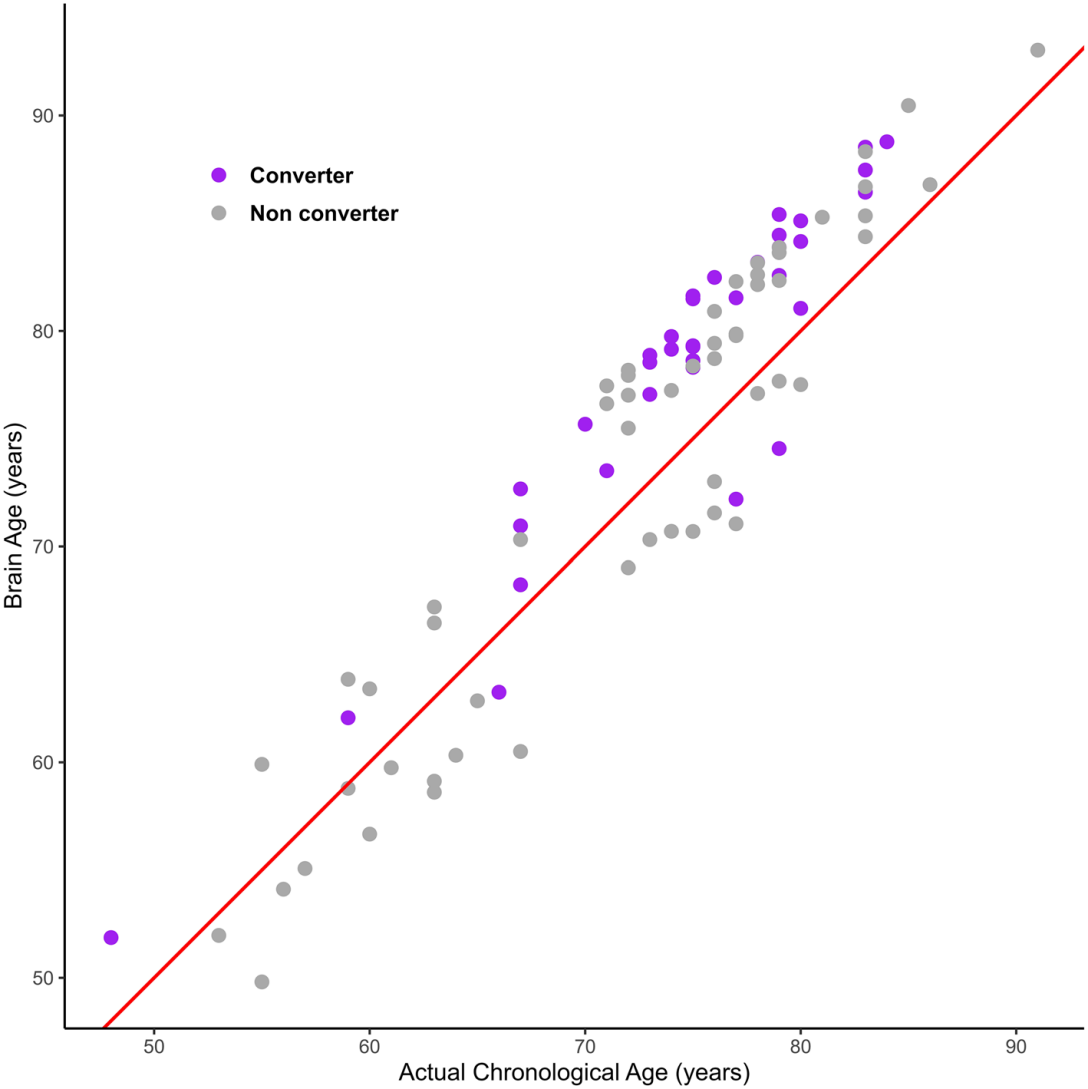
Comparison of brain age with the actual chronological age. The red line is y = x. In the conversion group (purple dots), 91% (32/35) had a brain age older than their actual chronological age (y > x). The conversion rate to dementia was significantly lower in the group with a younger brain age than their actual age (y < x).

We compared the brain age with the actual chronological age of the patients. In the conversion group, all participants except three (91%, 32/35) had a brain age greater than their chronological age (Fig. 2, purple dots). Patients in the non-conversion group (*N* = 56) were comparatively diffusely scattered in the comparison graph (Fig. 2, gray dots). When the brain and actual ages were compared between the two groups, the group with a brain age less than their actual age had a significantly lower (p = 0.003) conversion rate to dementia from MCI. The age at onset, diagnosis, and MRI scans were significantly greater in the group with a greater brain age, and the K-MMSE score was significantly higher in the group with a less brain age (Supplementary Table S1).

### Statistical subgroup analysis

#### Statistical differentiation in brain age subgroup analysis

Participants were divided into four subgroups. First, by comparing the brain and actual ages, we defined the older group as the group with patients having a brain age greater than their actual age, and the younger group as group with patients having a brain age less than their actual age. Second, converters and non-converters were separated. Finally, the formed four groups were as follows: group 1, younger by brain age- non-converters (*N* = 22); group 2, older by brain age non-converters (*N* = 34); group 3, younger by brain age converters (*N* = 3); and group 4, older by brain age converters (*N* = 32). As the number of patients in group 3 was very small, we decided to analyze the differences and compare the other three groups, that is, groups 1, 2 and 4. For continuous variables that did not show a normal distribution, the Kruskal–Wallis test was used. After performing the Kruskal–Wallis test, a post hoc test was performed using Benjamin Hochberg's p-value-adjusted method. Age, sex, and education were not adjusted because the effect of age, sex, and education level on each test was 0 or the effect on the test in each group was not the same. As shown in Supplementary Table S2, there were significant differences in the K-MMSE, CDR-SOB, RCFT delayed, RCFT recognition, COWAT animal, and ideomotor apraxia scores between the groups.

In this study, discrimination between older by brain age groups, that is, groups 2 and 4, was the primary mode for determining whether the patient will develop to dementia. The conversion rate in the older by brain age group was 48.5% (32/66), which differed significantly from the ’lower conversion rate in the younger by brain age group (12%, 3/25) as described earlier. The CDR-SOB, RCFT delayed, RCFT recognition, and COWAT animal test scores differed significantly between groups 2 and 4. The K-MMSE scores and other test domain scores did not differ between the two groups. The test results or past histories did not differ significantly between the non-converters and groups 1 and 2.

#### Survival analysis results of conversion

**Figure 3:**
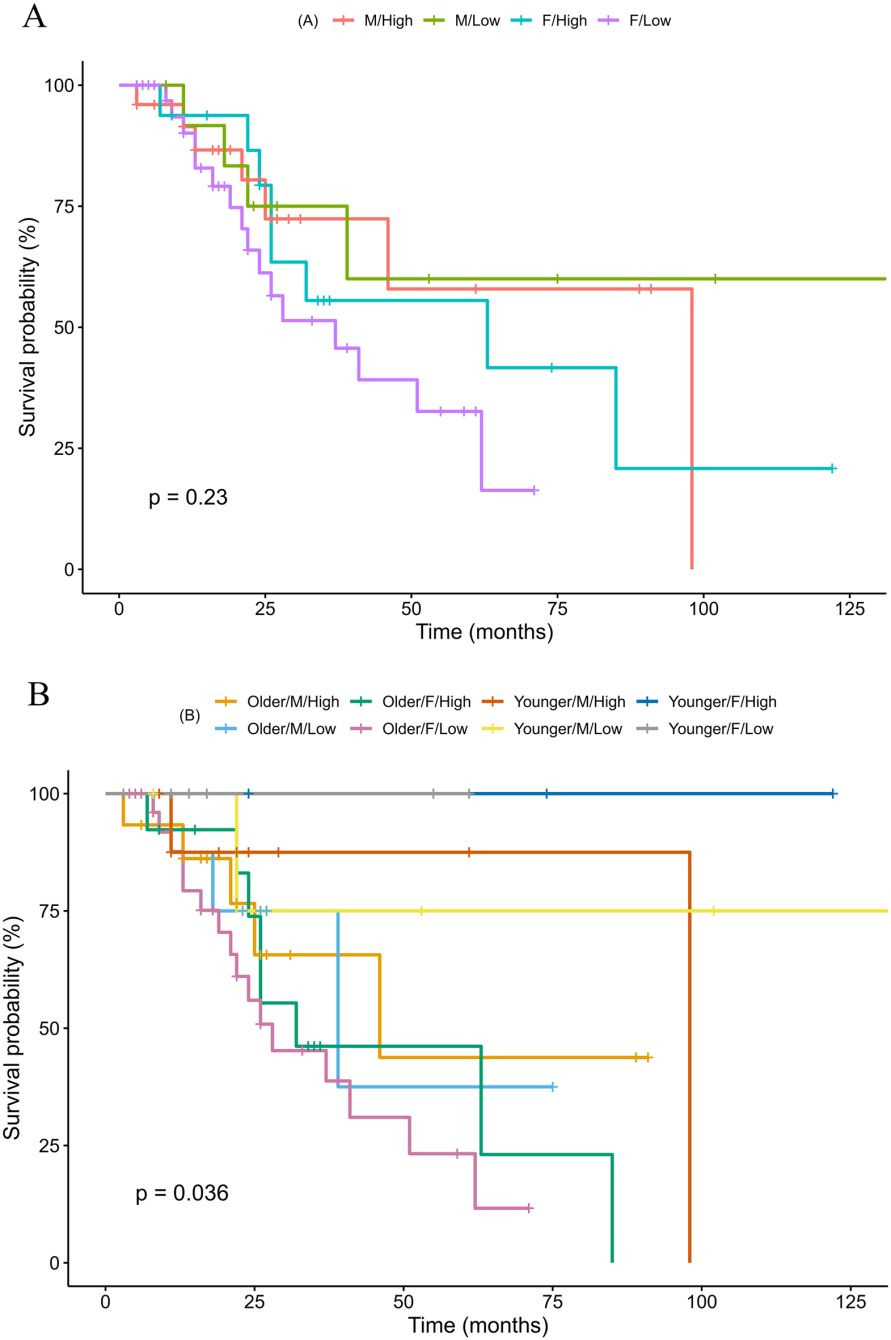
Survival analysis results of conversion. The graph demonstrates the interval survival rate at each event point during the entire study period and ultimately calculate the cumulative survival rate. The ‘survival' is defined as the non-conversion of MCI to dementia. A. The four groups, divided by sex and education level, did not significantly differ in survival probability with a p-value of 0.23. B. When groups were divided based on sex, education level, and brain age, eight groups significantly differed in survival probability with a p-value of 0.036. The low-educated female group with a higher brain age than their actual age (orange line) had the lowest survival rate of all groups. * ‘+' sign means censored data.

We defined the term ‘survival’ as the non-conversion of MCI into dementia and analyzed whether sex and education level affected survival probability. The division criteria were applied as follows: high level of education, education duration ≥ 12 years and low level of education, education duration < 12 years. There was no significant difference in the survival probability among the four groups formed according to sex and education level (p = 0.230, Fig. 3A).

When the participants were divided into eight groups according to sex, educational level, and the additional application of brain age division (older group, brain age > actual age; younger group, brain age < actual age), survival probability differed significantly among groups (p = 0.036; Fig. 3B). The low-educated female group with a brain age greater than their actual age (Fig. 3B, pink line) had the lowest survival rate among all groups.

## Discussion

Brain age calculated using quantitative information provided by the brain MRI AI software could be a novel imaging marker for predicting dementia conversion in patients with amyloid-negative amnestic MCI. Our study provides further evidence on the potential clinical utility of brain age prediction in identifying individuals at risk for dementia as the implication of four noticeable findings.

The first and main result of this study was that the older by brain age group had a 6.9 (CI 1.88–25.31) fold higher dementia conversion rate than the younger by brain age group (Supplementary Table S3). Patients with MCI having a brain age older than their actual age had heterogeneous features in terms of conversion. Hence, such patients should have regular clinical checkups for at least 36 months, which is the follow-up period of this study.

Second, the actual age at onset, diagnosis, and MRI scans did not differ significantly between converters and non-converters (Table 1), indicating that the effect of actual age was insignificant in these specific patient groups in terms of dementia conversion. Patients of all 3 age groups were significantly older in the brain age-older group compared to the brain age-younger group (Supplementary Table S1). As actual age may affect the cortical volume included in the formula for calculating the brain age, these results were expected^18^. However, because the values of ROIs are already adjusted for the same sex and age before including in the formula, brain age does not always correlate with actual age, as expected. These findings support the idea that brain age could be another prognostic marker that differs from actual age in patients with amyloid-negative amnestic MCI^19,20^. In addition, other factors, such as sex, education level, and ApoE genotype, did not differ between the two groups. However, we noticed that the subtracted value from brain age to age at the time of the MRI scan was significantly higher in the converter group. This finding indicates that the gap between the two types of ages can serve as a predictor of conversion of MCI to dementia (Supplementary Fig. S1).

Third, we found prominent frontoparietal dysfunction in converters in the older by brain age group. These findings indicate the need for careful assessment of frontoparietal dysfunction as an indicator of dementia conversion in amyloid-negative amnestic patients with MCI. Non-converters of older and younger by brain age groups did not have any significantly different features, indicating that brain age has a lower impact on the discrimination of non-converting patients.

Lastly, the brain age calculated by the model in this study helped discriminate survival probability, while sex and education level did not affect the estimation of dementia conversion, as shown in Fig. 3. The results suggested that the low-educated female group with a brain age greater than their actual age (Fig. 3, pink line) had the lowest survival rate of all groups, partially concurring with previous studies, indicating that dementia conversion is highest in the low-educated female group^21,22^. However, the finding that the younger by brain age, low-educated female group had a very high survival probability (Fig. 3, pink line) implies that brain age should be used as an additional predictive index in estimating dementia conversion^23^. As the number of patients in each subgroup was small, further evaluation with a larger sample size is warranted to validate this result.

Although our model shows feasible results for use in the neurological and neuroradiological fields, further research is needed to determine its potential clinical utility and to establish guidelines for its use in the diagnosis and management of dementia.

In this study, confirmation of pathological mechanisms other than amyloid was not performed. As amyloid PET follow-up study was also not performed. From enrollment, other cause of neurodegenerative disease has been excluded, thus, tau, TAR DNA-binding protein 43 (TDP-43), hippocampal sclerosis, and argyrophilic grain disease might still be the cause of these specific disease group. Furthermore, investigation on the influence of social and lifestyle factors including physical activity, smoking, and alcohol consumption is warranted. These variables may play a significant role in explaining the interindividual variability in brain age, extending beyond the impact of education alone.

In conclusion, our findings suggest that brain age using quantitative information provided by the brain MRI AI software can contribute to predicting conversion to dementia in patients with amyloid-negative amnestic MCI.

## Methods

### Ethical approval

We declare that all methods were performed in accordance with the relevant guidelines and regulations. The Institutional Review Board (IRB) of the Asan Medical Center waived the need for informed consent for the study. And the protocol of this study was also approved by IRB of the Asan Medical Center (#2019-0738). The study was performed in accordance with relevant guidelines and regulations. Also, we state that no live animals were used in this study.

### Participants

A total of 211 patients with amyloid-negative amnestic MCI with the following inclusion criteria who visited the memory clinic of Asan Medical Center from March 2013 to March 2016 were recruited: (1) age over 50 years with at least a 36-month follow-up period; (2) MCI defined by the criteria proposed by Petersen^24,25^; and (3) no visual evidence of amyloid deposition in amyloid positron emission tomography (PET) scans.

**Figure 4:**
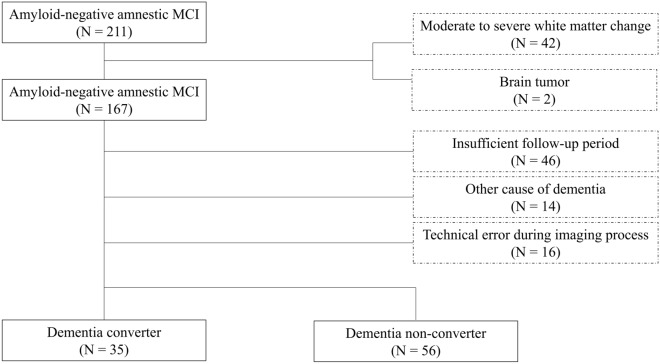
Patient disposition. Flow chart for this study from the initial screening to the final analysis. The solid outline squares represent the subjects that remained. The dash line squares represent the excluded subjects. Abbreviation: MCI, mild cognitive impairment.

Patients with the following exclusion criteria were excluded: (1) stroke, brain tumors, or white matter changes greater than a modified Fazekas scale score of 2 were excluded from the dataset (*N* = 42); (2) a history of traumatic brain injury, seizure, or current systemic medical illness (*N* = 2); (3) other causes of dementia such as Parkinson’s disease, corticobasal syndrome, diffuse Lewy body dementia, idiopathic normal pressure hydrocephalus, or frontotemporal dementia (*N* = 14); and (4) follow-up period less than 36 months (*N* = 46). All diagnostic processes were performed approximately 3 months after the neuropsychological tests. Additionally, 16 patients were excluded from the dataset because of technical errors caused by MRI artifacts during the imaging process. Therefore, the final sample comprised 91 patients with amyloid-negative amnestic MCI (Fig. 4).

### Cognitive measurement and diagnosis of amnestic MCI

All patients were evaluated using the Seoul Neuropsychological Screening Battery (SNSB) as a formal test, which is a comprehensive neuropsychological battery that includes tests for assessing attention (forward/backward digit span), language (comprehension, repetition, confrontational naming, reading, and writing), calculation, praxis (buccofacial and ideomotor), visuospatial function (Rey Complex Figure Test [RCFT]), verbal memory (Seoul Verbal Learning Test assessing immediate recall, delayed recall, and recognition), visual memory (RCFT assessing immediate recall, delayed recall, and recognition), and frontal/executive function (contrasting program, go/no-go test, verbal fluency, and the Stroop test). We also performed several other clinical and cognitive performance measurements, including the Korean version of the Mini-Mental State Examination (K-MMSE), Global Deterioration Scale (GDS), Clinical Dementia Rating (CDR), Neuropsychiatric Inventory, and Geriatric Depression Scale.

All patients visited the clinic regularly at intervals of 3–6 months and were interviewed by neurologists. The point of dementia conversion was determined by a clinical interview with a skilled neurologist using detailed neuropsychological evaluation, including assessment using the Seoul-Instrumental Activities of Daily Living (ADL) scale. For patients who did not undergo neuropsychological evaluation, an experienced neurologist determined their status based on a decline in K-MMSE scores of more than 4 per year with definite evidence of dysfunction in instrumental ADL (use of public transportation, shopping independently, and banking).

MCI was diagnosed based on changes in patients' cognition, objective evidence of impairment in one or more cognitive domains, preservation of independence in ADL. Similar to our previous study^26^, only patients with amnestic MCI were included in this study. The amnestic subtype was determined on the basis of scores below the 16^th^ percentile (–1 standard deviation) for demographically matched norms in verbal and visual memory tasks. Patients with both single- and multiple-domain amnestic MCI were included.

### Imaging acquisition

MRI was performed using a 3.0-T system (Achieva; Philips Medical Systems) with a sensitivity-encoding, eight-channel head coil. A high-resolution anatomical three-dimensional (3D) volume image was obtained using a 3D gradient-echo T1-weighted sequence with the following parameters: repetition time/echo time, 9.9/4.6 ms; flip angle, 8°; field of view, 224 × 224 mm; matrix, 224 × 224; slice thickness, 1 mm with no gap.

All PET images were obtained using Discovery 690, 710, and 690 Elite PET/CT scanners (GE Healthcare, Milwaukee, WI, USA). Amyloid PET images were acquired for 20 min, beginning 90 min after the injection of 300 ± 30 MBq of [^18^F] florbetaben. PET images were assessed using a predefined Brain Amyloid Plaque Load (BAPL) scoring system. The final score was determined by consensus among 2 skilled nuclear medicine specialists and 1 neurologist, with BAPL1 being Aβ-negative and BAPL2 and BAPL3 being Aβ-positive. Only patients with BAPL1 expression were included in this study.

### Formula for calculation of brain age

In this study, we introduce a novel formula to estimate brain age using brain volume data, which provides an age estimate that is lower than the actual age for cognitively normal individuals and higher than the actual age for those with cognitive abnormalities. Our approach relies on the premise that cerebral atrophy serves as a reliable marker of declining neurobiological health, and we achieve precise measurements of distinct brain regions by utilizing commercially available AI-driven segmentation algorithms^27,28^.

**Figure 5:**
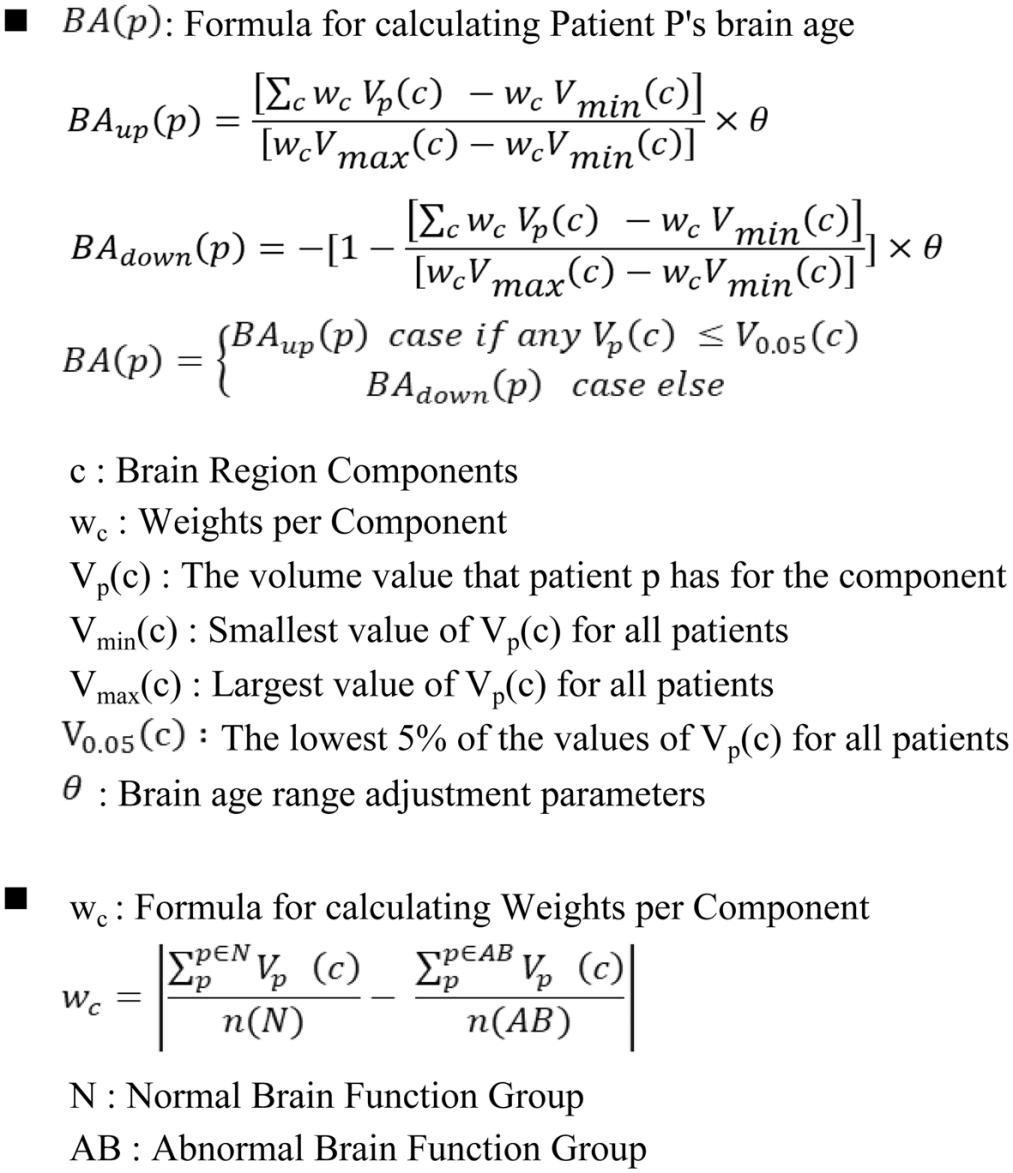
Calculation formula of the brain age.

We specifically identified and quantified regions of interest (ROIs) within the total brain volume, following the provided formula to calculate brain age (Fig. 5). Our selection of these ROIs was based on the identification of 12 regions (Supplementary Table S4) which showing significant volume differences, confirmed by t-tests between individuals with cognitive abnormalities such as MCI and dementia, and those with normal cognition.

Calculating brain age involves several steps: measuring volumetric differences in regions of interest (ROIs) between cognitively unimpaired (CU) and cognitively impaired (CI) groups. Weight values (w_c) quantify ROI volume differences for each component (c). Patients with dementia are in the CI group. ROI volumes are multiplied by w_c and summed to compute Brain age (BA) for each patient. This process varies based on the presence of an "atrophic region," defined as a bottom 5 percentile volume area for a specific ROI. An atrophic region results in an older brain age (BA_up), while its absence yields a younger age (BA_down). Brain age is calculated using age gap (g(p)) and a scaling parameter (θ), typically set to 7, providing a brain age estimation ranging from 7 years younger to 7 years older than the actual age.

### Statistical analysis

To compare the effectiveness of using the brain age index to distinguish between the dementia conversion and non-conversion groups, we analyzed the collected data with and without the brain age index using the statistical methods described below.

First, we examined the distribution of each group variable by conducting normality tests (Shapiro–Wilk test) and homogeneity of variance tests (Levene test). Equivalence tests of the regression coefficients were performed to determine the presence of interactions, to assess the need for demographic adjustments in the comparison tests. Based on the results of these tests, we selected appropriate statistical methods to assess group differences. For normally distributed data with equal variance, two-sample independent t-tests were used. Welch’s t-test was used for normally distributed data with unequal variance. For non-normally distributed data, we used the Kruskal–Wallis test. For categorical variables, we used the chi-squared test. If more than three groups were compared, we used the Kruskal–Wallis test. Spearman’s rank correlation coefficient was used to assess the correlation between the volume of the brain region used to calculate brain age and demographic or neuropsychological data.

In addition, we performed a survival analysis to investigate the difference in the rates of diagnosis of dementia between the groups, according to sex, education level, and brain age. The occurrence of dementia was regarded as an event, and data from patients who were not diagnosed with dementia within the 36-month follow-up period were considered censored data. The Kaplan–Meier estimation method was used to estimate the rate of event occurrence at the time of the event according to the observation time. We used the log-rank test to compare survival curves between groups and the Cox proportional hazard model to calculate the hazard ratio and significant variables.

All statistical analyses were performed using R software (version 4.2.2, R Foundation for Statistical Computing, Vienna, Austria), and the following R packages were used: car, stats, survival, survminer, and rms. Statistical significance was determined using a p-value threshold of 0.05.

### Supplementary Information

Supplementary Information.

## Data Availability

The data that support the findings will be available on request from the corresponding author. The data are not publicly available due to privacy or ethical restrictions.
